# Process modeling and bottleneck mining in online peer-review systems

**DOI:** 10.1186/s40064-015-1183-4

**Published:** 2015-08-22

**Authors:** Wichian Premchaiswadi, Parham Porouhan

**Affiliations:** Graduate School of Information Technology, Siam University, Bangkok, Thailand

**Keywords:** Online peer review system, Process discovery, Conformance checking, Performance analysis, Bottleneck mining, Social network analysis, LTL checker, Petri nets

## Abstract

This paper is divided into three main parts. In the first part of the study, we captured, collected and formatted an event log describing the handling of reviews for proceedings of an international conference in Thailand. In the second part, we used several process mining techniques in order to discover process models, social, organizational, and hierarchical structures from the proceeding’s event log. In the third part, we detected the deviations and bottlenecks of the peer review process by comparing the observed events (i.e., authentic dataset) with a pre-defined model (i.e., master map). Finally, we investigated the performance information as well as the total waiting time in order to improve the effectiveness and efficiency of the online submission and peer review system for the prospective conferences and seminars. Consequently, the main goals of the study were as follows: (1) to convert the collected event log into the appropriate format supported by process mining analysis tools, (2) to discover process models and to construct social networks based on the collected event log, and (3) to find deviations, discrepancies and bottlenecks between the collected event log and the master pre-defined model. The results showed that although each paper was initially sent to three different reviewers; it was not always possible to make a decision after the first round of reviewing; therefore, additional reviewers were invited. In total, all the accepted and rejected manuscripts were reviewed by an average of 3.9 and 3.2 expert reviewers, respectively. Moreover, obvious violations of the rules and regulations relating to careless or inappropriate peer review of a manuscript—committed by the editorial board and other staff—were identified. Nine blocks of activity in the authentic dataset were not completely compatible with the activities defined in the master model. Also, five of the activity traces were not correctly enabled, and seven activities were missed within the online submission system. On the other hand, dealing with the feedback (comments) received from the first and the third reviewers; the conference committee members and the organizers did not attend to those feedback/comments in a timely manner.

## Background

Currently, online and blind peer review of manuscripts submitted by authors is an important part of the publication process. However, a peer review does not necessarily include the assessment of a work by one or more reviewers who are experts in that field of study only. It is a process that includes selection and invitation of expert reviewers, allocation of deadline for reviewers, collection of comments from reviewers, discussion of manuscripts in detail, analysis of reviewers’ comments by an editorial board, invitation of more reviewers, a decision to accept or reject the manuscript, notification to authors about decisions, and so on. Typically, the most difficult part of a peer review process is selecting appropriate reviewers. The invited reviewers may be too busy with other commitments and obligations and are not able to participate in any manuscript revision tasks. On the other hand, some reviewers may initially show interest to take part in a peer review process but later refuse to provide any feedback or comments concerning the assigned manuscript. The main objective of the study was to investigate and scrutinize the peer review process of an international conference in a private university in Thailand. To do this, we initially developed an online reviewing system that: (1) could automatically store and acknowledge the online submissions, (2) could assign independent reviewers for the submitted manuscripts, (3) could easily show the status of the submission during the review, (4) could indicate whether a reviewer has participated in the reviewing process or not, (5) could automatically collect feedbacks from invited reviewers, (6) could send the received comments and feedback to the editorial board for making a decision, (7) could invite more reviewers and repeat the same steps if necessary, (8) could accept or reject a manuscript based on the final decisions made by the editorial board, and (9) could record and extract all of the above mentioned steps in terms of datasets.

In this study, we aimed to apply two classes of process mining techniques (i.e., Discovery and Conformance Analysis) in order to discover models, organizational structures, and bottlenecks related to the handling of proceedings’ peer reviews in an international conference in Thailand. Knowing that process mining analysis tools receive the input logs only in MXML (Mining eXtensible Markup Language) and XES (eXtensible Event Stream) formats, we initially converted the collected event log into a MXML-formatted log. Later, Alpha (α) Algorithm, Heuristic, Fuzzy and Social Network mining techniques (from Discovery class) were used in order to automatically construct the proceedings’ review models based on the authentic data and without having any priori model. Though the Heuristic Miner looked much similar to the Alpha algorithm, the technique had the privilege to better deal with XOR and AND connectors based on the dependency relations of the event log. Next, the actual process behavior was projected onto fuzzy models. The result was an animation movie which helped us to better understand the real activities occurred (during the proceeding’ peer review process). Furthermore, by using Social Network Miner technique we aimed to analyze the organizational perspective of the peer review process in terms of three metrics, namely as: (a) Handover of Work, (b) Working Together, and (c) Similar Tasks (Aalst 2011).

On the other hand, we aimed to monitor the deviations by comparing the observed events (real-life data) with the predefined models, as well. Having a priori model for review of the proceedings, we used LTL Checker and Performance Analysis techniques (from Conformance Checker class) to identify discrepancies between the log and the pre-defined model. After applying the LTL checking approach, the discrepancies and deviations were detected—leading to enrich the real model. In addition, Performance Analysis technique made us capable of projecting the bottlenecks all through the peer review system. Table 1 illustrates some of the process mining terms and techniques (supported by ProM 5.2) used in this study.

**Table 1:** Process mining techniques and terms used in this study

Plug-in	Description
*Alpha miner*	Discovers a Petri net using the *α*-algorithm
*Heuristic miner*	Discovers a C-net using heuristic mining
*Fuzzy miner*	Discovers a fuzzy model using fuzzy mining
*Social network miner*	Creates a social network based on a selected criterion
*LTL checker*	Checks a property expressed in terms of LTL
*Fitness*	Computes fitness of Petri net based on event log
*Replying*	The process of checking, comparing and connecting traces of events in an authentic log with a pre-defined master model
*Conformance checker*	Analyzes the gap between a model and the real data to detect violations
*Performance analysis with petri net*	Uses replaying to retrieve various Key Performance Indicators

Source: (Aalst 2011).

In general, one of the main benefits of the techniques used in this paper is that information is objectively compiled. To say simple, we gathered valuable information about what actually was happening according to the review process of the proceedings’ peer review process and existing bottlenecks, and not what we just thought or expected to see happening in the event log. Considering the results of the study, conference committee chairs and organizers can better evaluate the performance of the involving reviewers (as well as the staff) within the assigned tasks. This will improve the performance, efficiency and effectiveness of the handling of reviews for prospective academic conferences.

### ProM and PromMimport framework

**Fig. 1:**
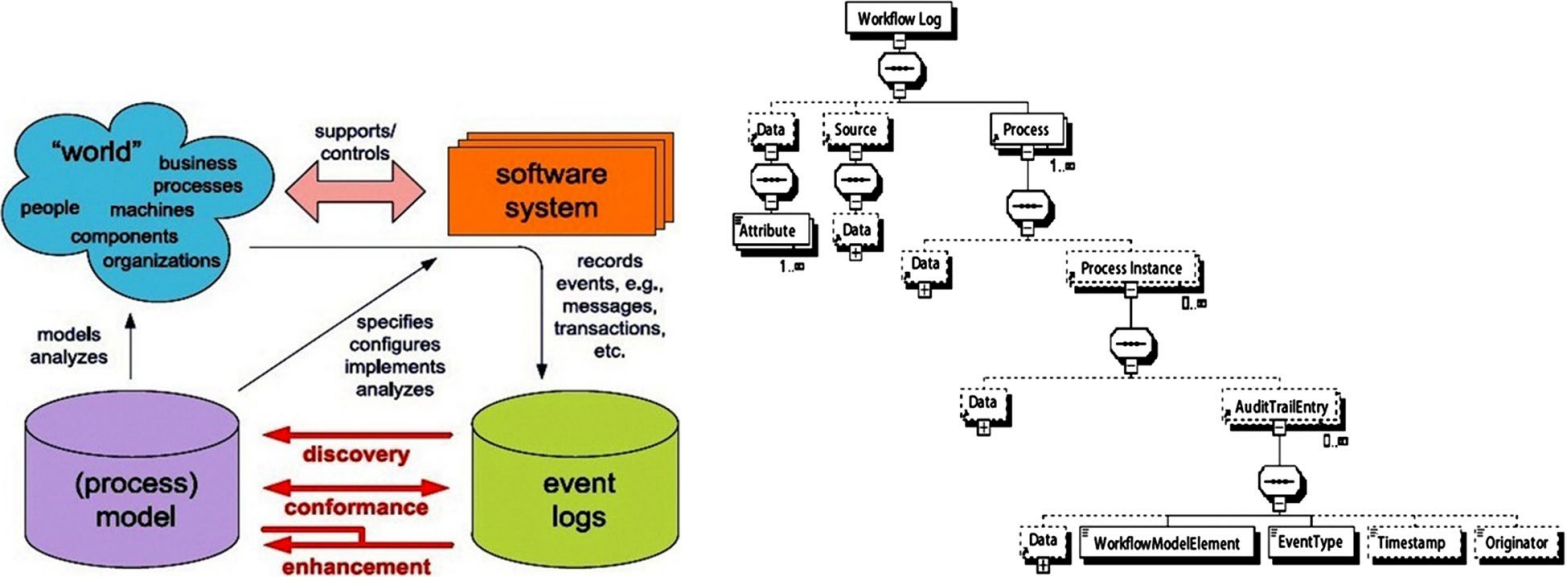
(*Left*) General process mining model. Three classes of process mining techniques include discovery, conformance and enhancement (Aalst 2011). (*Right*) MXML is one of the standard formats for storing event logs as an input format for ProM (Source: Aalst et al. 2007; Aalst 2011).

ProM is a generic framework for implementing process mining tools in a standard environment. The ProM framework^a^ receives the input logs in XES or MXML format. Currently, this framework has plug-ins for process mining, analysis, monitoring and conversion. The ProM framework has been developed as a complete plug-able environment. It can be extended by simply adding plug-ins and currently more than 90 plug-ins have been added (Krinkin and Kalishenko 2013). Figure 1 (left) illustrates how process mining plug-ins can be categorized. The plug-ins that are based on the data in the event log are called discovery plug-ins because they do not use any existing information about deployed models. The plug-ins that check how much data in the event log matches the prescribed behavior in the deployed models are called conformance plug-ins. Finally, the plug-ins called extension plug-ins need both a model and its associated logs to discover information that will enhance this model (Chang et al. 2008). It should be noted that *ProMimport*^b^ can be used to import event logs from various systems (e.g., Staffware and FLOWer) such that they can be analyzed using ProM (Aalst et al. 2007). Figure 1 (right) illustrates the standard MXML (Mining eXtensible Markup Language) format. The *ProcessInstance* elements correspond to cases. One *ProcessInstance* element may hold multiple *AuditTrailEntry* elements. Each of these elements represents an event. Each *AuditTrailEntry* element may contain *WorkfowModelElement*, *EventType*, *Timestamp*, and *Originator* elements. The *WorkfowModelElement* and *EventType* are mandatory elements (Aalst and Hee 2002; Aalst et al. 2007; Dumas et al. 2005).

## Related work

The idea of process mining is not new. Cook and Wolf (1996) conducted many research projects in the context of software engineering processes. Initially, they described three methods for process discovery: one using neural networks, one using a purely algorithmic approach, and one using a Markovian approach. The authors considered the latter two as the most promising approaches. The purely algorithmic approach built finite state machine where states were fused if their *futures* (i.e., in terms of possible behavior in the next k steps) were identical (Cook and Wolf 1996). The proposed Markovian approach used a mixture of algorithmic and statistical methods and was able to deal with noise in simple process maps. However, their results were limited to sequential behavior. Cook and Wolf (1998) extended their work to concurrent processes. They proposed specific metrics—such as entropy, event type counts, periodicity, and causality—and used these metrics to discover models within event streams. However, they did not provide an approach to generate explicit process models. Later, Cook and Wolf (1999) provided a measure to quantify discrepancies between a process model and the actual behavior as registered using event-based data.

The thought of applying process mining in the context of workflow management was first introduced in the sixth International Conference on Extending Database Technology held at Valencia, Spain (Agrawal et al. 1998). This work was based on the workflow graphs which were inspired by work flow products such as IBM MQSeries workflow (formerly known as Flowmark) and InConcert (Zhang 2010). In 2001, a tool based on these algorithms was made available (Maxeiner et al. 2001).

Schimm (2000, 2002) developed a mining tool suitable for discovering hierarchically structured workflow processes. This required all splits and joins to be balanced. Herbst and Karagiannis (1998, 1999) also addressed the issue of process mining inthe context of workflow management using an inductive approach. Although, their work was limited to sequential models; their proposed approach allowed for concurrency analysis. Herbst (2000) used stochastic task graphs as an intermediate representation using a workflow model described in the ADONIS modeling language. In the introductory step, task nodes were merged and split in order to discover the underlying process. Weijters and Aalst (2001a, b) developed a heuristic approach to construct so-called “dependency/frequency tables” and “dependency/frequency graphs”. However, the preliminary results presented in their work only provided heuristic approaches and focused more on issues such as noise. Aalst and Dongen (2002) presented the EMiT tool which used an extended version of α-algorithm^c^ to incorporate timing information.

Some other studies (Burt and Minor 1983; Aalst et al. 2004; Scott and Carrington 2011) used sociometry (also referred to as sociography) as a method to present data on interpersonal relationships in a graph or matrix form. The term sociometry was coined by Jacob Levy Moreno (Moreno and Jennings 1934) who conducted the first long-range sociometric study at the New York State Training School for Girls in Hudson, New York (Wasserman and Faust 1994; Song 2004). As part of this study, Moreno and Jennings (1934) used sociometric techniques to assign residents to various residential cottages. They found that assignments on the basis of sociometry substantially reduced the number of runaways from the facility. Since the early work of Moreno and Jennings, sociometry, and Social Network Analysis^d^ in particular, have been active research domains (Desel et al. 2004). Workflow management systems like Staffware register the start and completion of activities (Aalst and Dongen 2002). ERP systems like SAP log all transactions, e.g., users filling out forms, changing documents, etc. Business-to-business (B2B) systems log the exchange of messages with other parties (Dumas et al. 2005). Call center packages as well as general purpose CRM systems log interactions with customers. These examples show that many systems have some type of event log often referred to as “history”, “audit trail”, “transaction file”, etc. (Aalst 2005).

Rozinat and Aalst (2008) proposed an incremental approach to check the conformance of a process model and an event log. Their goal was the detection of inconsistencies between a process model and its corresponding execution log, and the quantification by the formation of metrics. Aalst et al. (2012) used ProM’s Conformance Checker^e^ and Performance Analysis with Petri Net^f^ tools—which are suitable for replaying—to establish a precise relationship between events and model elements. By applying these techniques, they could diagnose deviations from the modeled behavior, and identify the severity of each deviation. Accordingly, their results could properly detect and show bottlenecks within the processes.

### Methodology

**Fig. 2:**
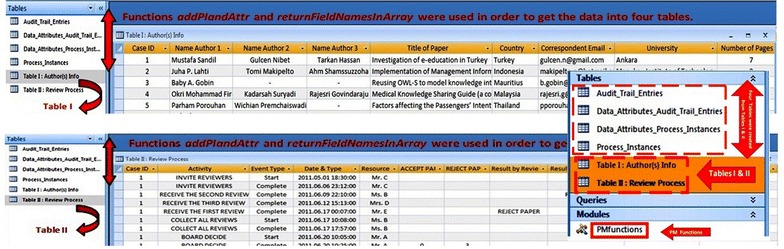
Three screenshots from the initial event log in MS Access Database format. The data from *Tables I* and *II* were extracted and then imported into four new tables namely as follows: process-instances table, data-Attributes-process-instances table, audit-trail-entries table, and data-attributes-audit-trail-entries table. In order to get the data into these tables, functions addPIandAttr and returnFieldNamesInArray were used in the Visual Basic script.

In this study, we used an event log describing the handling of reviews for an international conference at a private university in Thailand. As shown in Fig. 2, the collected event log was originally in *Microsoft Access Database* format. The event log consisted of 87 papers (referred to as cases) and 3267 events. The Editorial Board of the conference consisted of over 30 academic experts in the fields of computer science, information technology and software engineering. After receiving a manuscript, each paper was sent to *three different reviewers* using an online and automatic handling system. It was not always possible to make a decision after the first round of reviewing a manuscript including reviewer 1, reviewer 2 and reviewer 3; therefore, if there was not enough feedback received from the invited reviewers, more reviewers were invited to participate. This process was repeated until a final decision could be made by Committee Members. The chair of the conference committee was responsible for making a decision whether to “accept”, “invite another reviewer”, or “reject” an article (after consulting with other members). In order to “accept” a paper, at least 2 out of 3 of the reviewers (or 67% of the collected feedback) needed to contain positive comments regarding a submitted manuscript. Alternatively, in order to “reject” a paper, at least 2 out of 3 of the reviewers (or 67% of the collected feedback) needed to contain negative comments about the submitted manuscript. Due to the fact that the event log contained personal and sensitive information concerning the authors’ names, place of the birth, e-mail address and so forth, we deliberately made anonymous some necessary parts of the event log (such as originators names) before starting any process modeling or bottleneck mining analysis. Table 1 shows some of the process mining terms and techniques (supported by ProM 5.2) used in this study.

**Fig. 3:**
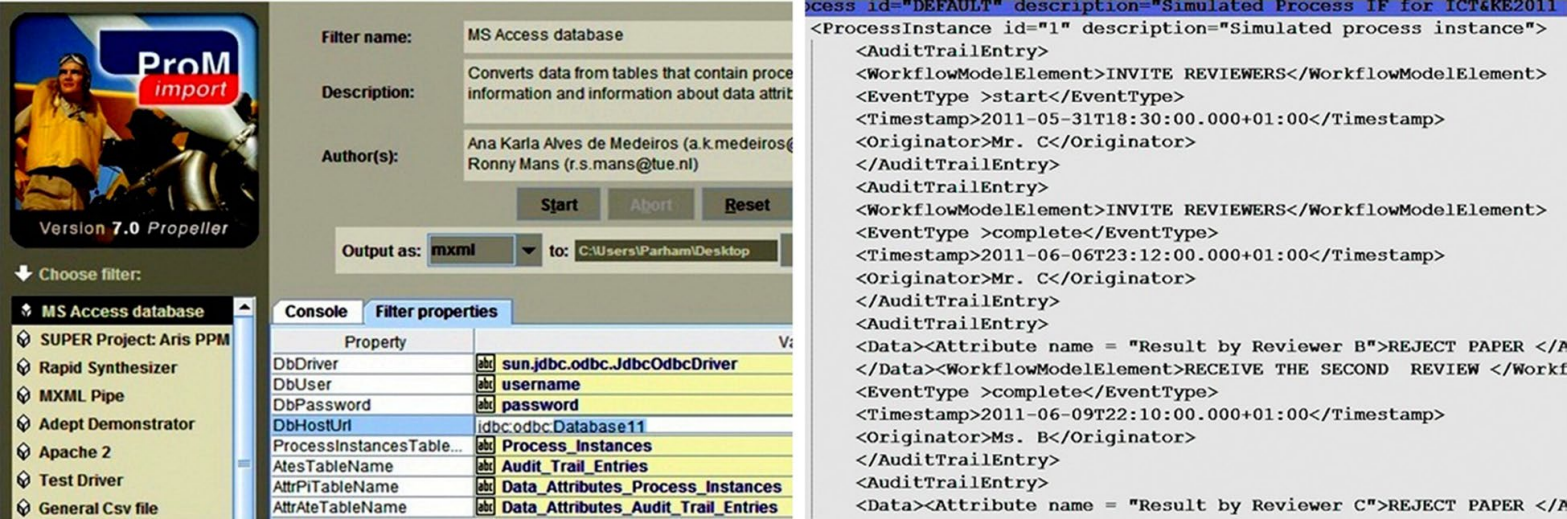
(*Left*) After configuring an ODBC connection to PC, the ProMimport was used in order to extract a MXML-formatted log from the modified Database with four tables. (*Right*) A screenshot of the resulting MXML-formatted log created by the ProMimport.

Prior to extracting any information from the event log, the data needed be converted into MXML format because ProM 5.2 framework only receives the input logs in the MXML format (Porouhan and Premchaiswadi 2012). As shown in Fig. 2, in order to convert the Microsoft Access database to MXML format, four tables with certain structures were initially filled with the relevant data (Dongen et al. 2009). The data dealt with information about the papers that were reviewed for an international conference in Thailand. The first table named “*Process_Instances*” deals with the identifier of a certain process instance. The second data table named “*Attributes_Process_Instances*” deals with additional information about each process instance (data attributes). The third table named “*Audit_Trail_Entries*” deals with data about activities and tasks that were run during the execution of the process instance. And finally, the fourth table named “*Data_Attributes_Audit_Trail_Entries*” deals with data attributes about activities and tasks. In order to fill the above mentioned four tables with relevant data, the functions *addPIandAttr* and *returnFieldNamesInArray* in the Visual Basic script were used (Dongen et al. 2009). These functions^g^ were written based on the concept that every row in the table includes information about a specific process instance as well as additional information about the attributes of each process instance.1As shown in Fig. 2 and the pseudocode (1), using the *addPIandAttr* function; two new tables of “*Process_Instances*” and “*Data_Attributes_Process_Instances*” from Table 1 (i.e., Authors Info) were extracted and created. Accordingly, the “Case ID” was defined as *Process Instance* and the “colNames” was used for *Process Instance Attributes*. The “colNames” parameter was a reference to an array. Therefore, a combination of the function *returnFieldNamesInArray* and “colNames 1, −1″ means that three parameters (i.e. −1, 0, +1) from Table I were chosen and exported into the two new *Process*-*Instances* and *Data*-*Attributes_Process_Instances* tables. Similarly, a combination of the function *returnFieldNamesInArray* and “colNames6, -1” means that seven parameters from Table II were chosen and exported into the two new *Audit*-*Trail*-*Entries* and *Data*-*Attributes*-*Audit*-*Trail*-*Entries*tables. Therefore, four new tables based on Tables I and II were created and filled with the appropriate data. Subsequently, as shown in Fig. 3, the *ProMimport* tool was used in order to convert the dataset (with four tables) into MXML format.

## Results and discussion

As mentioned above, there are many different process mining techniques with respect to three basic classes: (1) process mining techniques for discovering new models, (2) process mining techniques for auditing and conformance checking of a model with real data, and (3) process mining techniques that can be used for enhancement purposes. This study was focused on the first and second classes of process mining techniques (i.e., Discovery and Conformance Analysis). From the discovery class, Alpha algorithm, Heuristic Mining, Fuzzy Mining, and Social Network Analysis techniques were applied in order to *discover* models and organizational structures related to the handling of the proceedings’ peer reviews for an international conference in Thailand. From the conformance analysis class, LTL Checker and Performance Analysis techniques were used in order to compare, check, and audit the authentic dataset with a pre-defined model. Accordingly, the main goals of the study were: (1) to convert the collected event log into the appropriate format supported by process mining analysis tools, (2) to discover process models and to construct social networks based on the collected event log, and (3) to find deviations, discrepancies and bottlenecks between the collected event log and the master pre-defined model. Therefore, the results of the applied approaches—considering the second (2) and third (3) goals of the study—are discussed as the following:

### Alpha (α) algorithm

**Fig. 4:**
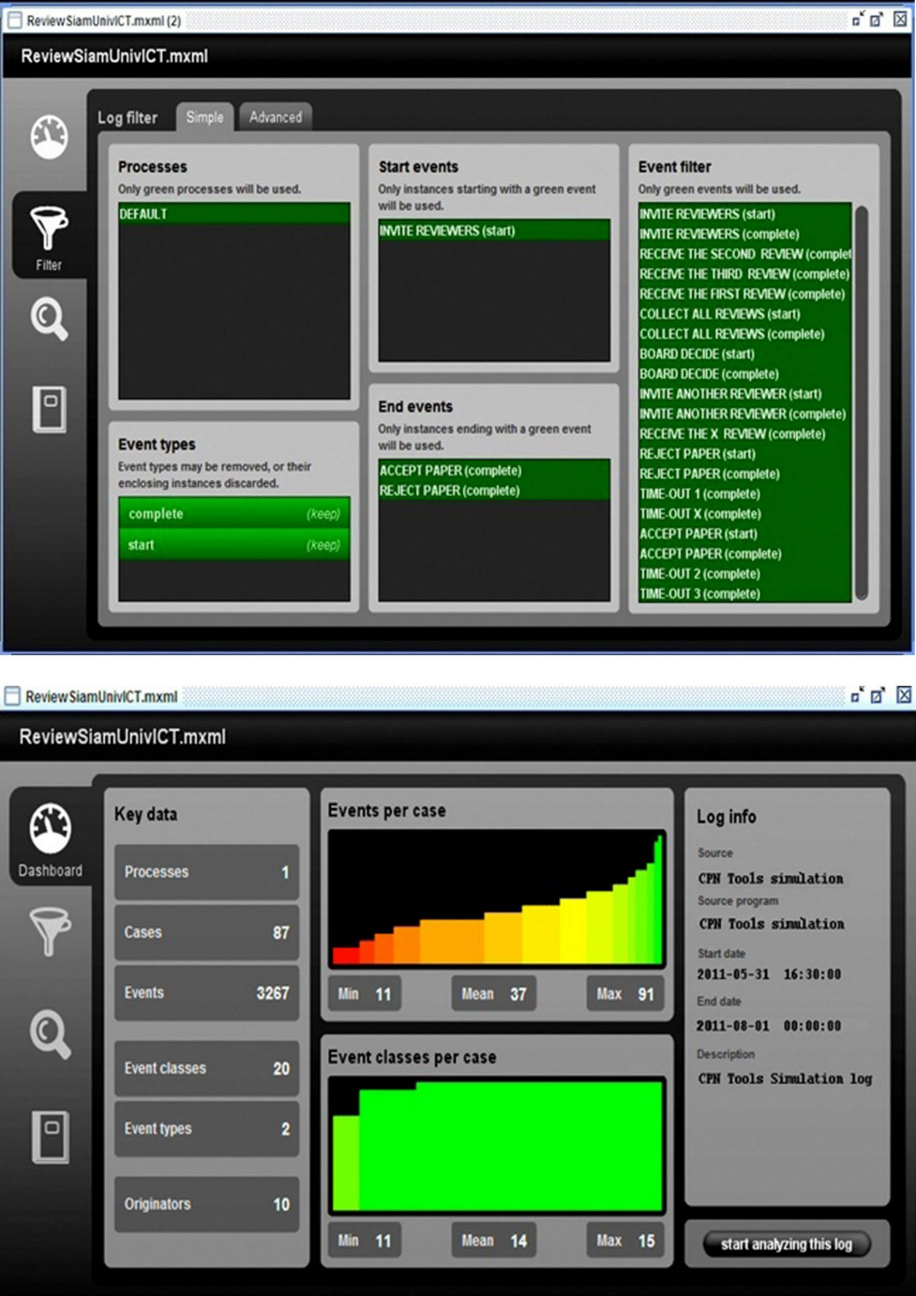
(*Up*) Using ProM’s Controls and Dashboard enabled us to better monitor the handling of peer-review, decision making, reviewer reports, and other tasks in the international conference in Thailand. (*Down*) A screenshot of the Log Summary of derived from the international conference’s proceeding review event log/dataset.

The main benefit of applying the α-algorithm in process mining is to reconstruct causality from a set of sequences of activities. The algorithm is capable of mining the control flow perspective of a process with respect to Petri Nets which deal with Place and Transition Nets (Burattin et al. 2014). The Alpha algorithm was first developed by Professor Wil van der Aalst from Technische Universiteit Eindhoven (TU/e) in The Netherlands (Devi and Sudhamani 2013). In this paper, we applied the Alpha algorithm as a technique to identify the routing constructs within the proceedings review system. Since our main emphasis was on the peer review process as a whole; therefore, we based our discovery on the “completed” process instances only. As a result, our log contained only two process types: Start and Complete. As shown in Fig. 4 (up), filtering the MXML event log allowed us to select only those types of events (i.e., tasks or audit trail entries) that we were interested in considering during the peer review process. In Fig. 4 (down), a general summary of the proceedings review process regarding the event log is illustrated. Since the peer review process in the international conference starts with inviting reviewers for reviewing the manuscripts, and ends with an accept, or a reject decision based on the board decision about the submitted manuscript; therefore, the process instance of “Invite Reviewers” was chosen as the starting event point while both process instances of “*Accept Paper*” or “*Reject Paper*” were chosen as the ending event points.

**Fig. 5:**
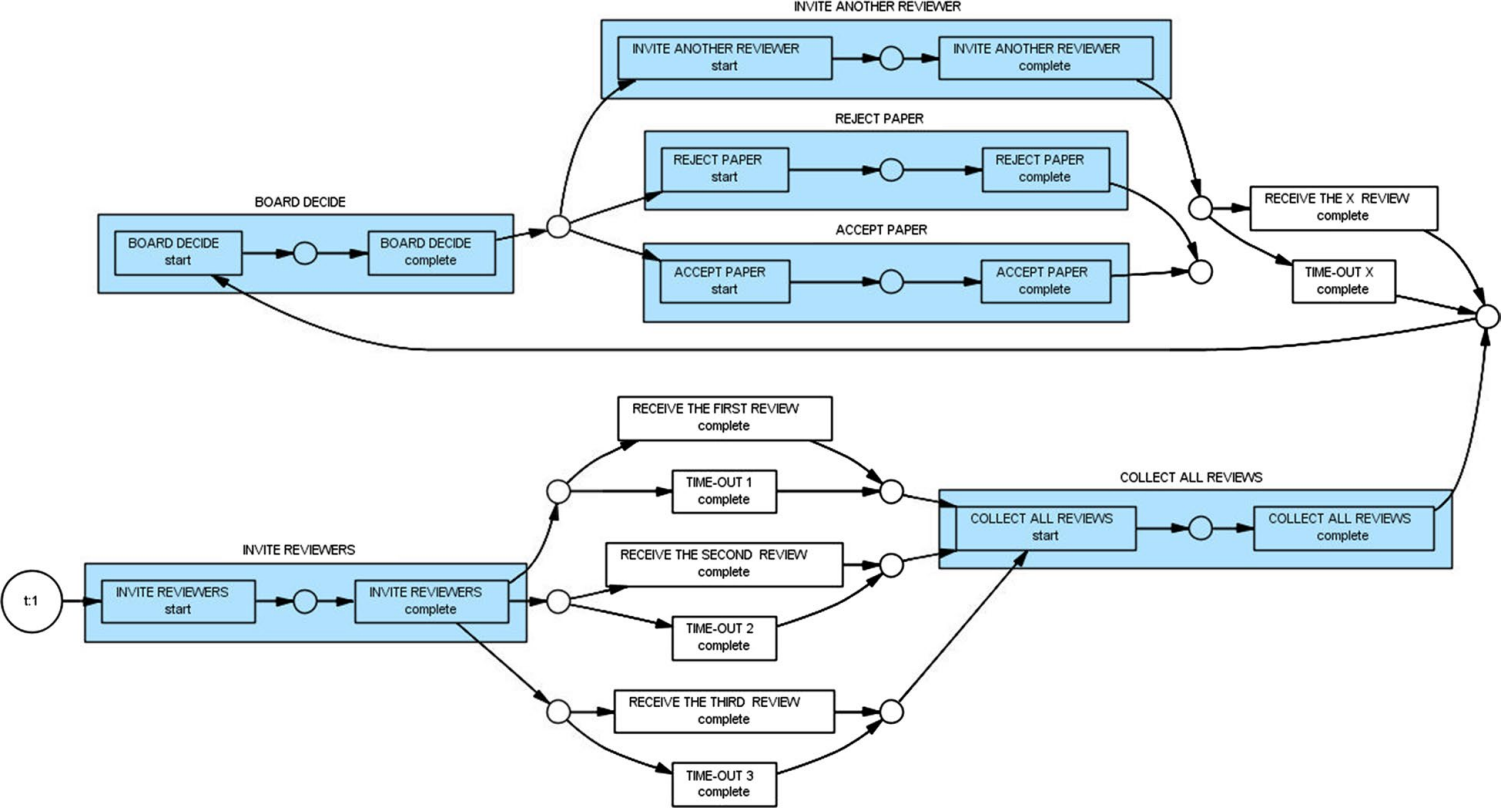
Using the Alpha-algorithm enabled us to create a process model that summarizes the peer-review flow followed by most cases in an international conference in Bangkok, Thailand (Aalst 2011).

Figure 5 shows a screenshot of the resulting model created by the Alpha algorithm based on the international conference’s peer-review event log. By studying the model, we are able to better investigate the peer review process with respect to: (1) the tasks that came before/after other tasks, (2) the tasks that concurrently occurred with other tasks, or (3) the tasks that were duplicated (i.e., loop) in the model.

### Heuristic mining

**Fig. 6:**
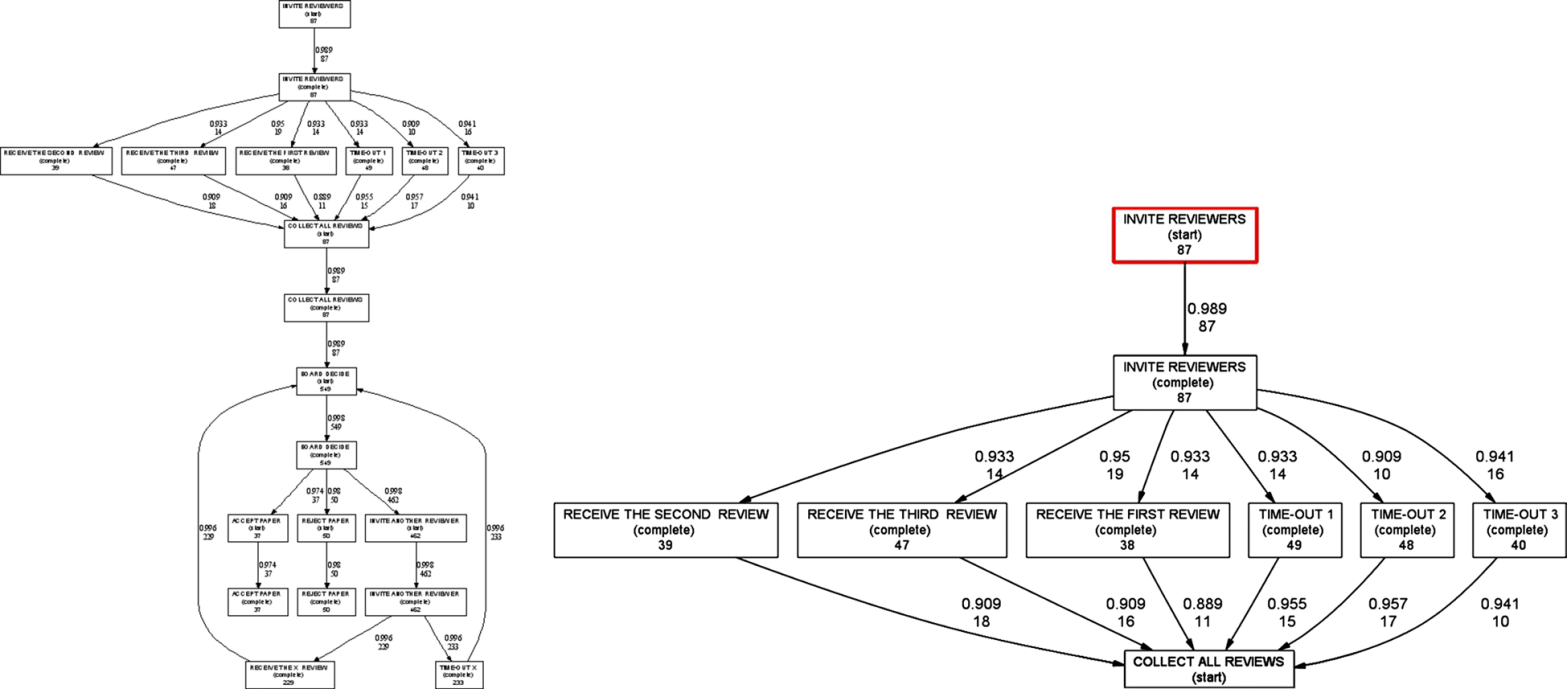
Two screenshots of the mined peer-review event log using the Heuristic Mining technique.

Although the resulting model created by the Alpha algorithm gave us a holistic view of the tasks executed during the peer review process, the produced model is not able to properly deal with noise in the log. Moreover, any frequency information about dependencies of the tasks was not taken into account by the Alpha algorithm. When dealing with noisy data, the Alpha algorithm does not necessarily produce reliable and robust models. Process maps and workflow nets may include several types of structures and constructs which the α-algorithm cannot rediscover (Aalst et al. 2004). If an event log contains short loops of length one, then α-algorithm is not capable of rediscovering them. Similarly, if an event log contains short loops of length two, α-algorithm is not capable of rediscovering them, either. And if an event log contains non-local dependencies, α-algorithm is not capable of properly dealing with process constraints. (Aalst 2011; Medeiros et al. 2004). Therefore, to avoid such constraints and to solve such problems, we applied the Heuristic Miner algorithm in order to look for causal dependencies where one task follows another task (i.e., Heuristic Miner algorithm was more sophisticated and adequate than α-algorithm). Our main objective was to create models that were less sensitive to the incompleteness of logs and contain noise. In Fig. 6, the rectangular boxes represent the activities or tasks; while the arrows indicate the dependency between activities, and the number in the event box shows the frequency of the activities that were performed. The number on the arrows shows the number of times the connection has been used. For example, the number 87 in the rectangular box of the activity “*Invite Reviewers*” shows that a total of 87 times the organizing committee members of the conference have invited a reviewer to review a manuscript. In the same manner, the number 19 on the arrow between the tasks “*Invite Reviewers*” to “*Receive The First Review*” indicates that the task “Receive The First Review” has been followed 19 times by the task “Invite Reviewers”. Again, number 14 on the arrow between the tasks “*Invite Reviewers*” to “*Receive The Third Review*” shows that the task “Receive The Third Review” has been followed 14 times by the task “Invite Reviewers”. The second number adjacent to the frequency number is called the *Dependency Measure* which indicates dependency relation between two tasks. A maximum value of 1.0 as a Dependency Measure represents the fact that there is a 100% full dependency relation between the connected tasks. Alternatively, a minimum value of 0 as a Dependency Measure represents the fact that there is no dependency relation between the connected tasks. However, although the resulting model created by the Heuristic Miner algorithm gave us a more sophisticated view (compared with α-algorithm) of the tasks executed during the proceedings’ peer review process, yet the produced model is not able to properly deal with mixed and complex AND and XOR joint/split situations. Moreover, if an event log contains complex spaghetti-like structures, then Heuristic Miner is not capable of rediscovering them. Similarly, if an event log contains dangling activities, missing connections, or missing activities, then the results of Heuristic Mining approach provide less meaningful information about the underlying processes. Therefore, a more sophisticated process discovery technique overcoming these limitations and constraints needed to be considered. In this paper, we found the Fuzzy Mining algorithm quite adequate (i.e., much more adequate than Heuristic Miner algorithm) as it does not only present visual process models but also properly deals with more complex structures which may exist within event logs (Saravanan et al. 2011).

### Fuzzy mining

**Fig. 7:**
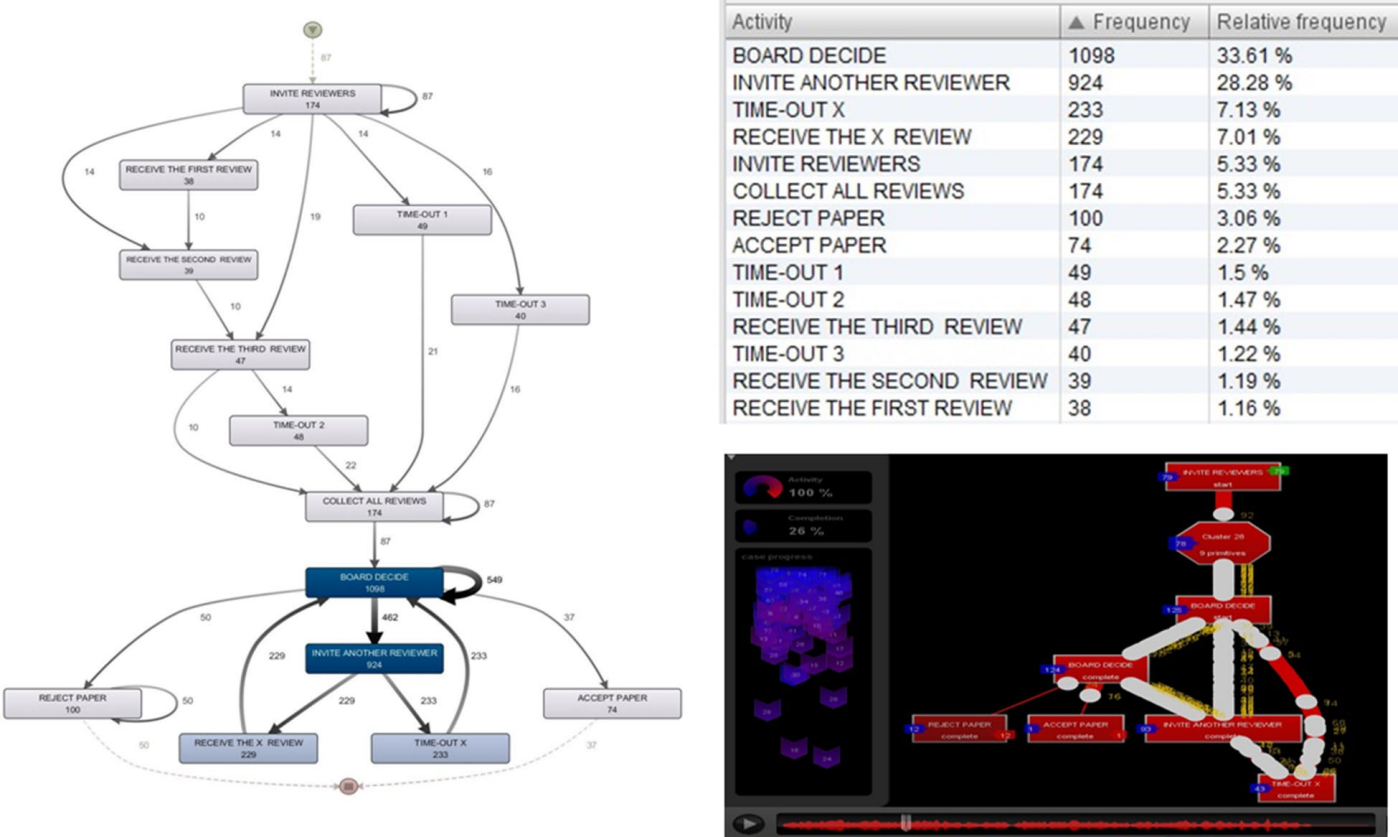
(*Left*) A screenshot of the Fuzzy Model generated by Fuzzy Mining algorithm. The *color* of each *rectangular box* represents the absolute frequency of times this task is executed. The thickness of each *arrow* represents the absolute frequency of times the path is taken. (*Right*) A screenshot of the resulting animation for the peer-review event log based on the Fuzzy Mining algorithm.

Process models should normally be able to present understandable and meaningful constructs of operational processes. Applying different process mining plugins, we sometimes encounter models that look very complex and meaningless without highlighting what is important in the data. Fuzzy mining is one of the traditional process mining techniques that is commonly used in order to deal with those types of complex models that are not easily comprehensible at the first glance. Figure 7 (left) shows a fuzzy model corresponding to the event log that was used to construct the process model of the proceedings review. The thickness of the arrows and the colors of the connections represent the level of absolute frequency of occurrence of the tasks, or the extent of relationship between the tasks (Premchaiswadi and Porouhan 2015). The produced fuzzy model shows all the activities as well as all the causal dependencies between them in a more sophisticated manner. When studying the resulting fuzzy model, we realized that the tasks “*Board Decide*”, “*Invite Another Reviewer*”, “*Time*-*Out X*”, and “*Receive The X Review*” were the most significant tasks (during the peer review process of the international conference) allocating 33.61%, 28.28%, 7.13%, and 7.01% of the performed tasks to themselves, respectively.

Using the Fuzzy Mining technique, we also could project all log traces onto a model simultaneously. Figure 7 (right) indicates an animation based on the historic information received from all log traces in the peer-review event log. The animation shows the actual execution of the cases (i.e., revision of manuscripts) based on the fuzzy model. The animation can be played multiple times in order to come up with a better understanding about what has really occurred during the peer review process in the international conference. Moreover, watching the animation helped the organizing committee members to differentiate individual process instances and observe the overall peer review process at a particular point in time. In addition, the technique significantly increases the awareness of the conference organizers about the parts that are more important (or less important) during the proceedings review process.

### Analysis of social network

The work presented in this paper investigated the *social network* (between the reviewers and the decision board members) with respect to three metrics as follows: (a) Handover of Task metric. This metric examined who passed a task to whom, (b) Similarity of Tasks metric. This metric examined who executed the same type of tasks and (c) Working Together metric. This metric examined how frequently individuals have worked on the same task.

#### Handover of task

**Fig. 8:**
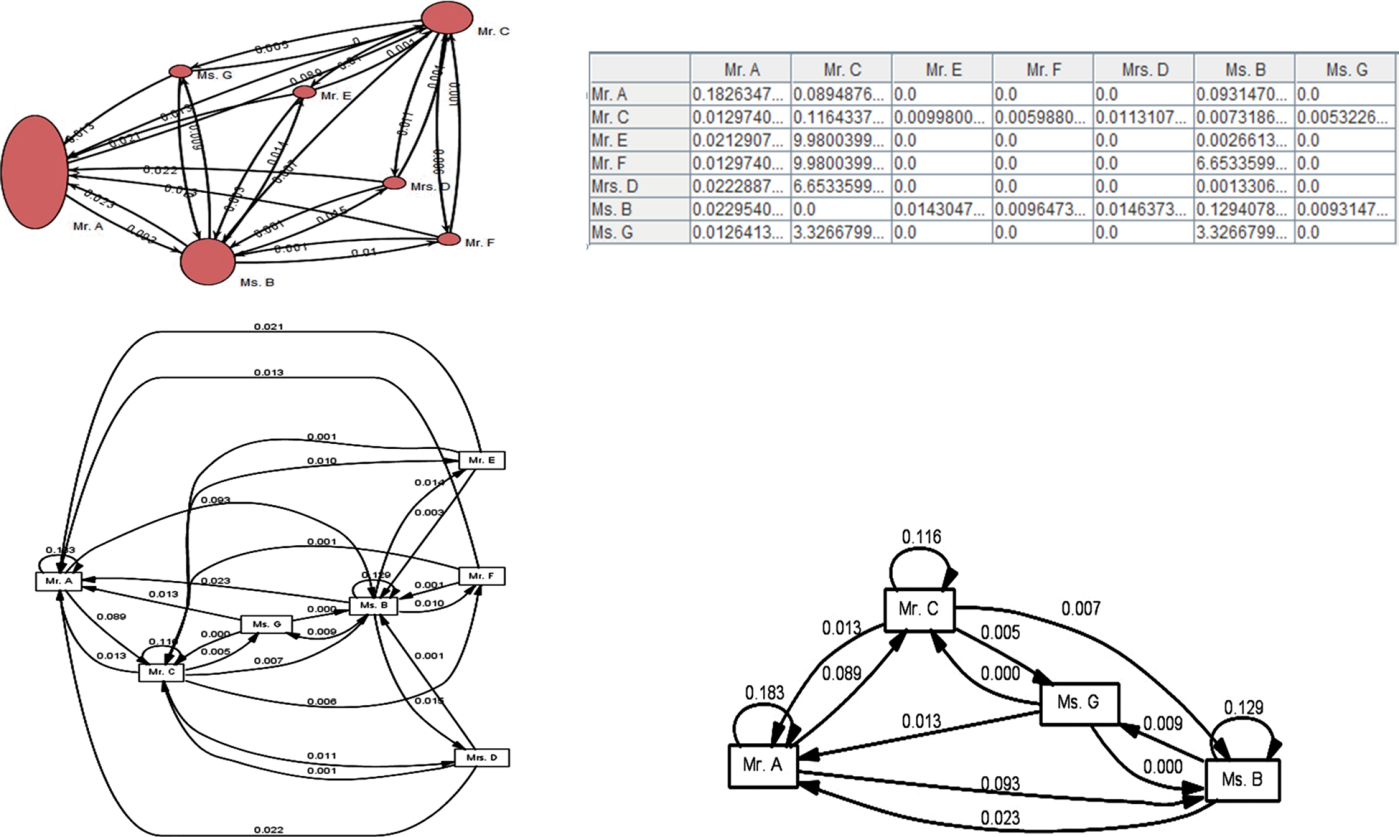
(*Up*) Two screenshots of the Social Network Analysis based on the Circle Layout and Hanover of Task matrix. The more the nodes receive tasks, the more vertical they look like. The more the nodes assign tasks to others, the more horizontal they look like. (*Down*) Two screenshots of the Social Network Miner (based on the Hanover of Task metric) on the peer-review event log. The more out-going *arrows* lead to appearance of the more horizontal *oval shapes.*

Within each process instance (i.e., Case), there is a handover of task from *Individual X* to *Individual Y* if there are two subsequent tasks where the first is completed by *Individual X* and the second by *Individual Y*. The graph in Fig. 8 (up) illustrates the Handover of Task graph for the conference committee members and the organizers (i.e., process instances). The nodes with oval shapes in the graph represent the relationship between the in and out extent of the exchanged tasks (shown with arrows). Thus, the more the nodes receive tasks, the more vertical they look like (or the more in-going arrows, the more vertical oval shapes appear). Alternatively, the more the nodes assign tasks to others, the more horizontal they look (or the more out-going arrows, the more horizontal oval shapes appear). Therefore, considering Fig. 8 (up) of the study, we realized that Mr. A and Ms. B were the most active (hardworking) members of the proceedings review process (i.e., few out-going arrows went out of their nodes). In other words, Mr. A and Ms. B received a large burden of work handed over from other members to them. Similarly, as shown in Fig. 8 (down), the number of in-going arrows coming to Mr. A and Ms. B are much greater, compared to the other conference committee members and organizers who receive fewer in-going arrows.

#### Similarity of task

**Fig. 9:**
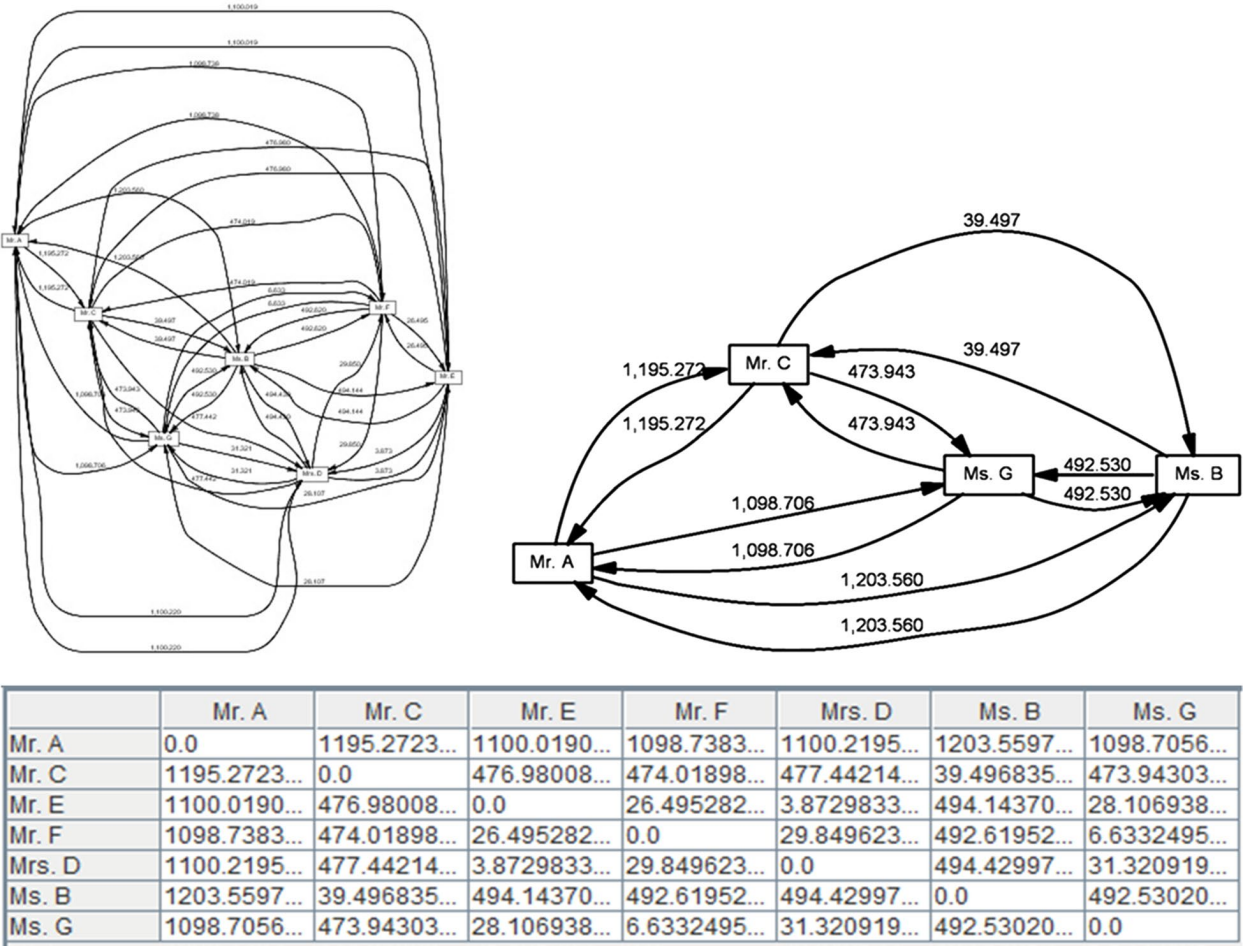
(*Up*) Two screenshots of the social network miner (based on the Similarity of task metric) on the peer-review event log. (*Down*) A screenshot of the similarity of task matrix.

Despite the Handover of Task metric, the Similarity of Task metric does not emphasize how activities were shared from one individual to another individual. This approach emphasizes the activities that individuals performed. In the Similarity of Task metric every individual has a profile based on the number of times he or she has performed specific tasks. There were many different approaches to determine the “distance” between 2 profiles. However, in this study we used a *similarity coefficient* for^h^ comparing the similarity of sample groups (Intarasema et al. 2012). As shown in Fig. 9, the graph clearly illustrates similar actions performed by Mr. A, Mr. C, Ms. B ad Ms. G. Normally individuals performing similar tasks have stronger relations than individuals performing completely different tasks.

#### Working together

**Fig. 10:**
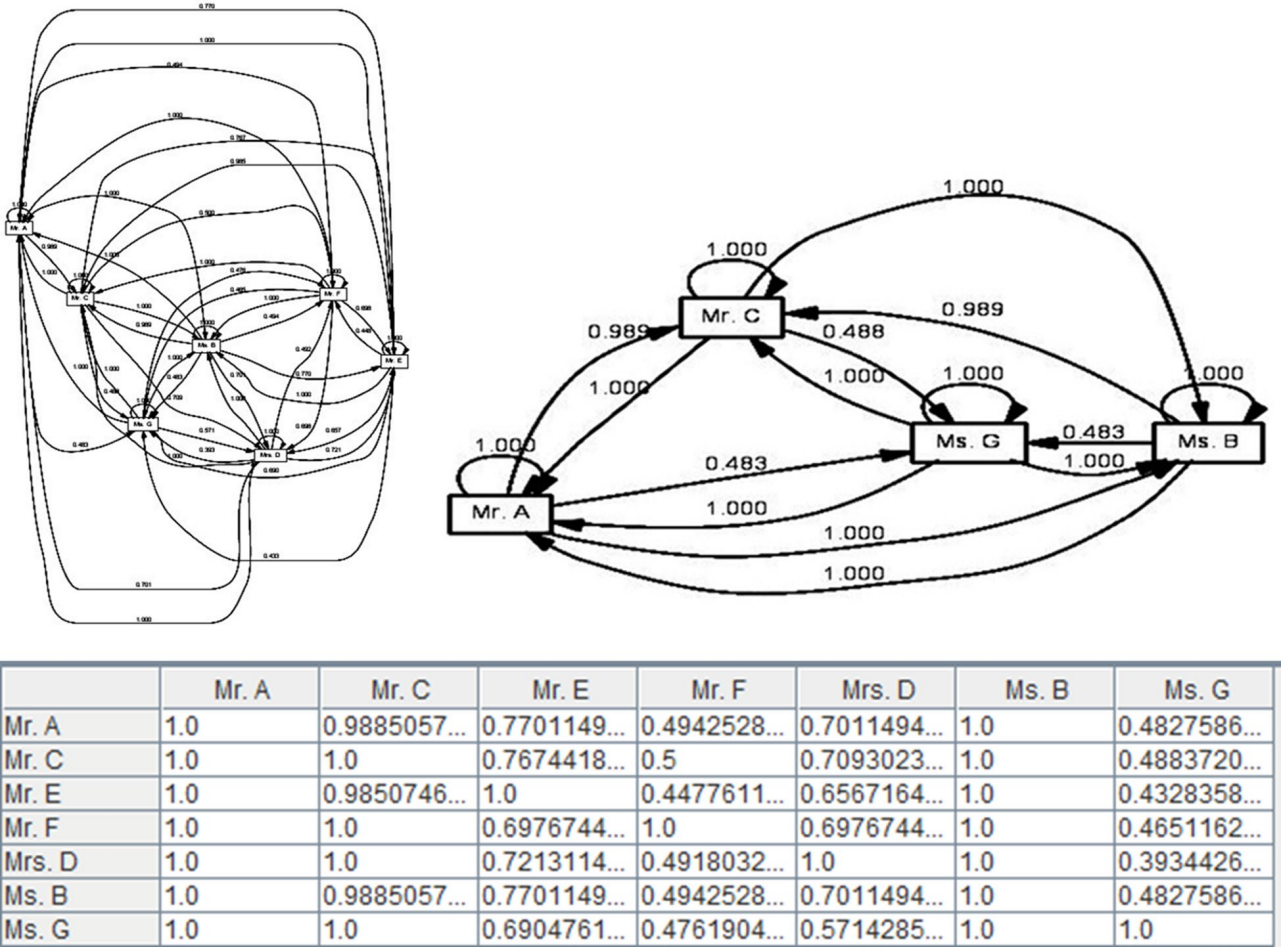
(*Up*) Two screenshots of the Social Network Miner (based on the Working Together metric) on the peer-review event log. (*Down*) A screenshot of the Working Together matrix using ProM Process Mining Framework.

Likewise, we wanted to know whether people in the same community work together or not. To address this issue, we focused on the cases (instead of the activities) using Social Network Miner technique. Figure 10 shows a social network obtained by the Working Together graph using ProM 5.2. The graph shows active participation and the collectiveness among Mr. A, Mr. C, Ms. B ad Ms. G. The Working Together graph is helpful when there are disjoint teams in the log.

#### Organizational mining

**Fig. 11:**
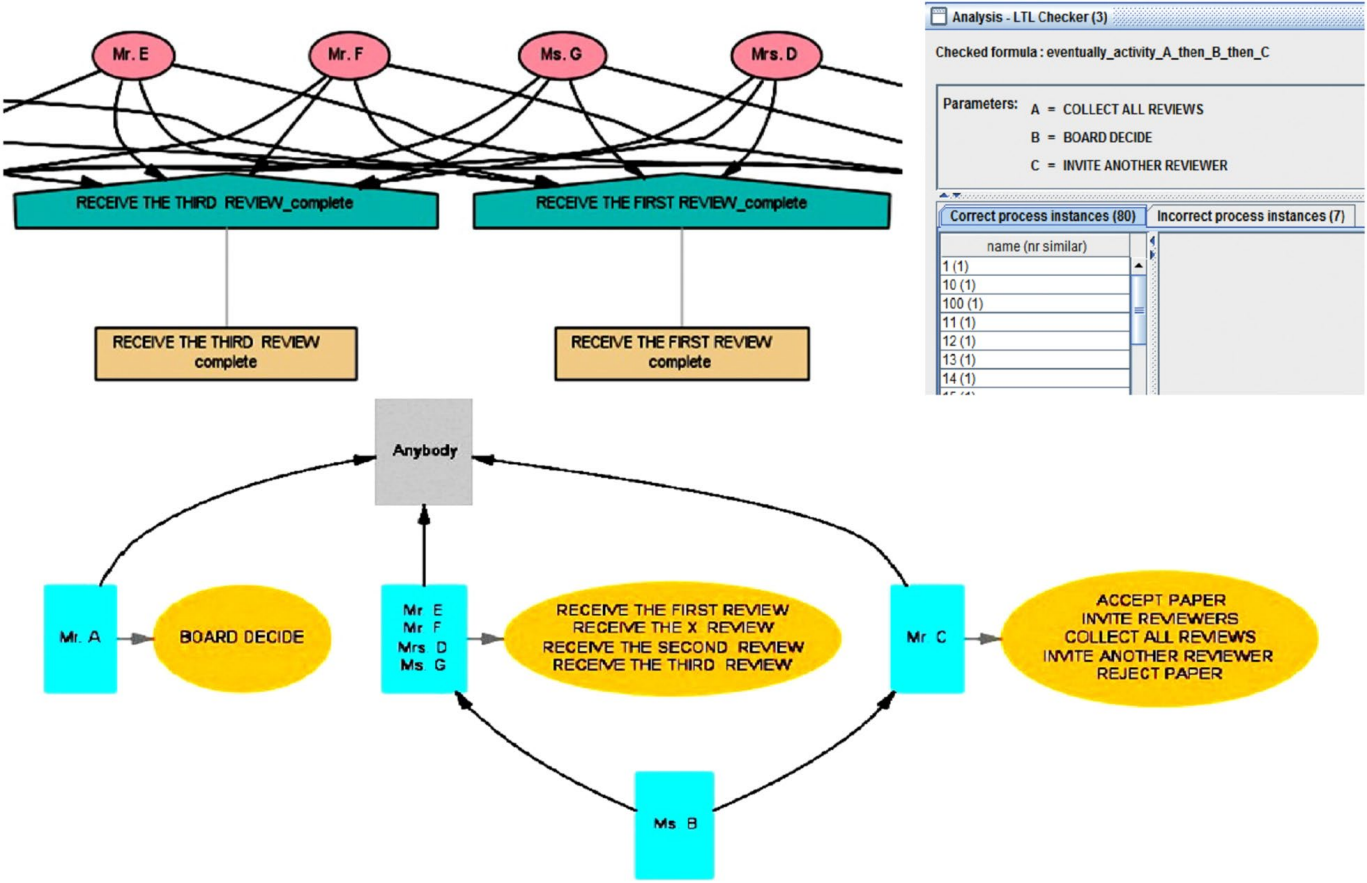
(*Up*) two small screenshot of the groups classified based on the Semantic Organizational Mining technique, as well as the results of Semantic LTL Checker approach for cases that followed the formula: eventually_activity A_then B_then C. (*Down*) A screenshot of the clusters generated based on the Role Hierarchy Mining technique using ProM Process Mining Framework.

Although the three previous techniques (i.e., Handover of Task, Similarity of Task, and Working Together) gave us interesting insights with respect to the relationships between individuals or tasks, these techniques are not able to provide information about the organizational structures within the event log. Therefore, we applied the Semantic Organizational Mining technique in order to investigate the peer review event log in terms of levels of structural organization. As shown on the left side of Fig. 11 (up), the semantic organizational miner technique enabled us to classify different groups of the conference committee members and organizers based on the similarity of tasks they performed. Tasks were assumed to be similar every time there were instances of the same concepts.

#### Role hierarchy mining

This approach is based on an Agglomerative Hierarchical Clustering technique which deals with joint activities in the peer review event log. Here, the main idea is to create multiple clusters which are consistent with the activities that each individual performs. Figure 11 (down) shows a dendogram derived from the Agglomerative Hierarchical Clustering technique. The technique enabled us to generate flat or disjoint organizational entities by adjusting the dendogram with a threshold (i.e., certain value). As a result, by adjusting the dendogram with a threshold value of 0, we obtained 4 different clusters (meaning there are 4 different groups of individuals with dissimilar roles and duties) within the peer review event log. The first group, cluster 1, included Mr. A as the Editor-in-Chief and head of the organizing committee. The second group, cluster 2, consisted of the Mr. E, Mr. F, Mrs. D, and Ms. G who were in charge of receiving the reviews/feedback from the invited reviewers. The third group, cluster 3, consisted of Ms. B who behaved as a medium between cluster 2 and Mr. C. Thus, Ms. B was constantly in touch with cluster 2 and Mr. C. And consequently, the fourth group, cluster 4, included Mr. C as the secretary of the conference in charge of inviting reviewers, collecting all the reviews, and announcing the board decisions to the authors (i.e., whether the manuscript is accepted or rejected). Therefore, using the Organizational Miner technique we could investigate the data at a higher level of abstraction—compared with the previously mentioned techniques. While the handover of task, similarity of task and working together metrics emphasized more on the individuals and the transactions between individuals; the Organizational Miner and Role Hierarchy Miner technique mainly focused on the teams, groups and hierarchies as a whole.

### Semantic LTL checker

It is often the case that processes for reviewing manuscripts need to obey specific rules and regulations. For instance, in order to make a decision about a manuscript (i.e., accept or reject) for the international conference, feedback (reviews) from at least three reviewers needed be received and collected. Similarly, any invitation of an additional reviewer was based on the board decision after careful investigation of the all initially collected reviews from reviewers. One way to check whether these rules and regulations have been actually obeyed is to audit the event log using certain techniques. We used the Semantic LTL Checker tool in ProM to verify and check the property: Does the task “*Invite Another Reviewer*” always happen after the tasks “*Board Decide*” and “*Collect All Reviewers*”? (i.e., formula: eventually_activity A_then B_then C). The resulting screen shown on the right side of Fig. 11 (up) indicates that the event log is divided into two main parts: (1) the part including the cases that satisfy the property (i.e., formula: eventually_activity A_then B_then C), and (2) the part including the cases that do not satisfy the property. Studying the LTL results for the correct process instances, we realized that in 80 of the cases (out of the total of 87 process instances) the task “*Invite another Reviewer*” eventually happened after “*Board Decide*” and after “*Collect All Reviews*”. However, in 7 of the cases (out of the total of 87 process instances) the task “*Invite another Reviewer*” did *not* happened after “*Board Decide*” and after “*Collect All Reviews*”. This is assumed to be an obvious violation of rules and regulations during the peer review process.

### Conformance checking

When implementing different process mining discovery algorithms or different social network analysis metrics, all of the process instances in the input event log are built and replayed based on the input Petri net. Therefore, it is possible that a process instance is not completely compatible (or does not fit) with the Petri net. In this section, we used the Conformance Checker technique in order to replay (i.e. audit) the peer review event log. The rationale behind this approach was to compare the level of fitness between the *authentic event log* and the *pre*-*defined master model*. Using this approach, when an activity is executed in a log trace; one of the matching transitions in the Petri net will be paired with that activity, and then the necessary measurements are taken.

#### Model perspective

We used the “*model perspective*” feature of ProM’s Conformance Checker plugin in order to replay the peer-review dataset with respect to: (1) number of missing activities (i.e., activities that were missed to be taken into account in the Petri net model), (2) number of failed activities (i.e., activities that were not enabled), (3) number of remaining activities (i.e., activities that remained enabled). The results showed that 9 blocks of activity in authentic peer review event log were not compatible/(no fit) with the activities in the pre-defined Petri net model (i.e., master model). Moreover, five activity traces were not correctly enabled in the authentic event log. Also, seven activities were missed in the authentic event log compared with the pre-defined master Petri net model. In other words, the resulting Petri net model and the real peer review event log were not a complete fit and compatible with each other at a few points and nodes.

#### Log perspective

**Fig. 12:**
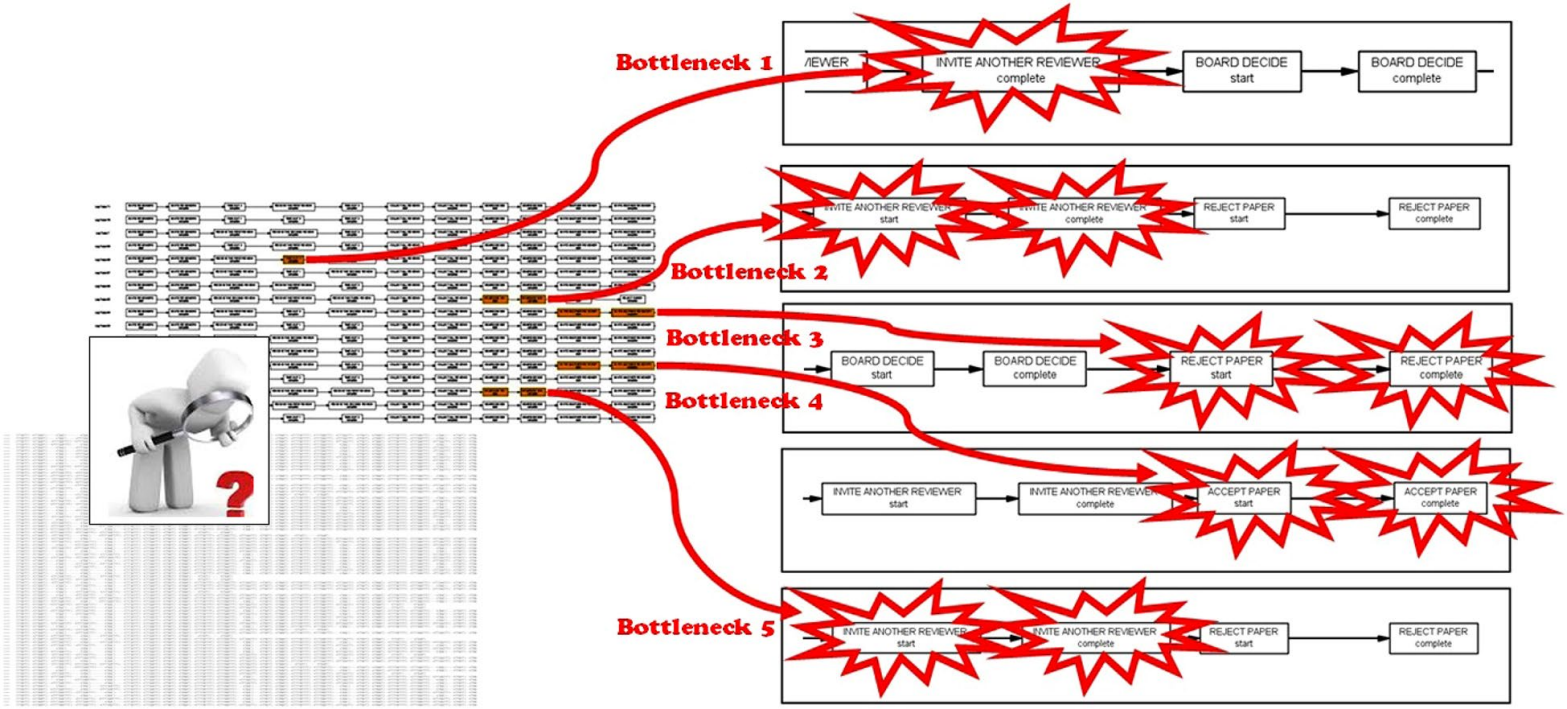
Using the Conformance Checking techniques (i.e., model perspective and log perspective) enabled us to detect discrepancies, bottlenecks and deviations of rules throughout the online peer-review process. The *figure* shows selected screenshots of the Conformance Checking (log perspective) results where problems were found based on the replaying method.

We also used the “*log perspective*” feature of ProM’s Conformance Checker plugin in order to check how much process instances in the peer review event log were compatible with the Petri net model. Our goal was to highlight the possible discrepancies and bottlenecks between the real log and the model. As shown in Fig. 12, the log perspective feature of the Conformance Checker approach indicated 9 blocks of activity (highlighted in orange color) that were not compatible with the pre-defined master Petri net model. Therefore, the violations and deviations during the peer review process for the international conference were identified as the following:

- *Bottleneck 1:* The board has made a decision before receiving feedback/reviews from Reviewer X. This is an obvious violation of the rules, as the board can only make a decision when feedback from a reviewer is received, or when the legitimate time for a reviewer to review the manuscript has ended.
- *Bottleneck 2:* The board has rejected a manuscript before receiving feedback from Reviewer X. This is an obvious violation of the rules, as the board can only reject a manuscript after receiving all of the feedback from all reviewers.
- *Bottleneck 3:* The board has rejected a manuscript after only receiving feedbacks from two reviewers. This is an obvious violation of the rules, as the board can only reject a manuscript after receiving feedback from at least three of the reviewers.
- *Bottleneck 4:* The board has accepted a manuscript before receiving feedback from Reviewer X. This is an obvious violation of the rules, as the board can only accept a manuscript after receiving all of the feedback from all reviewers.
- *Bottleneck 5:* The board has rejected a manuscript before receiving feedback from Reviewer X. This is an obvious violation of the rules, as the board can only reject a manuscript after receiving all of the feedbacks from all reviewers.

### Performance analysis with petri net

**Fig. 13:**
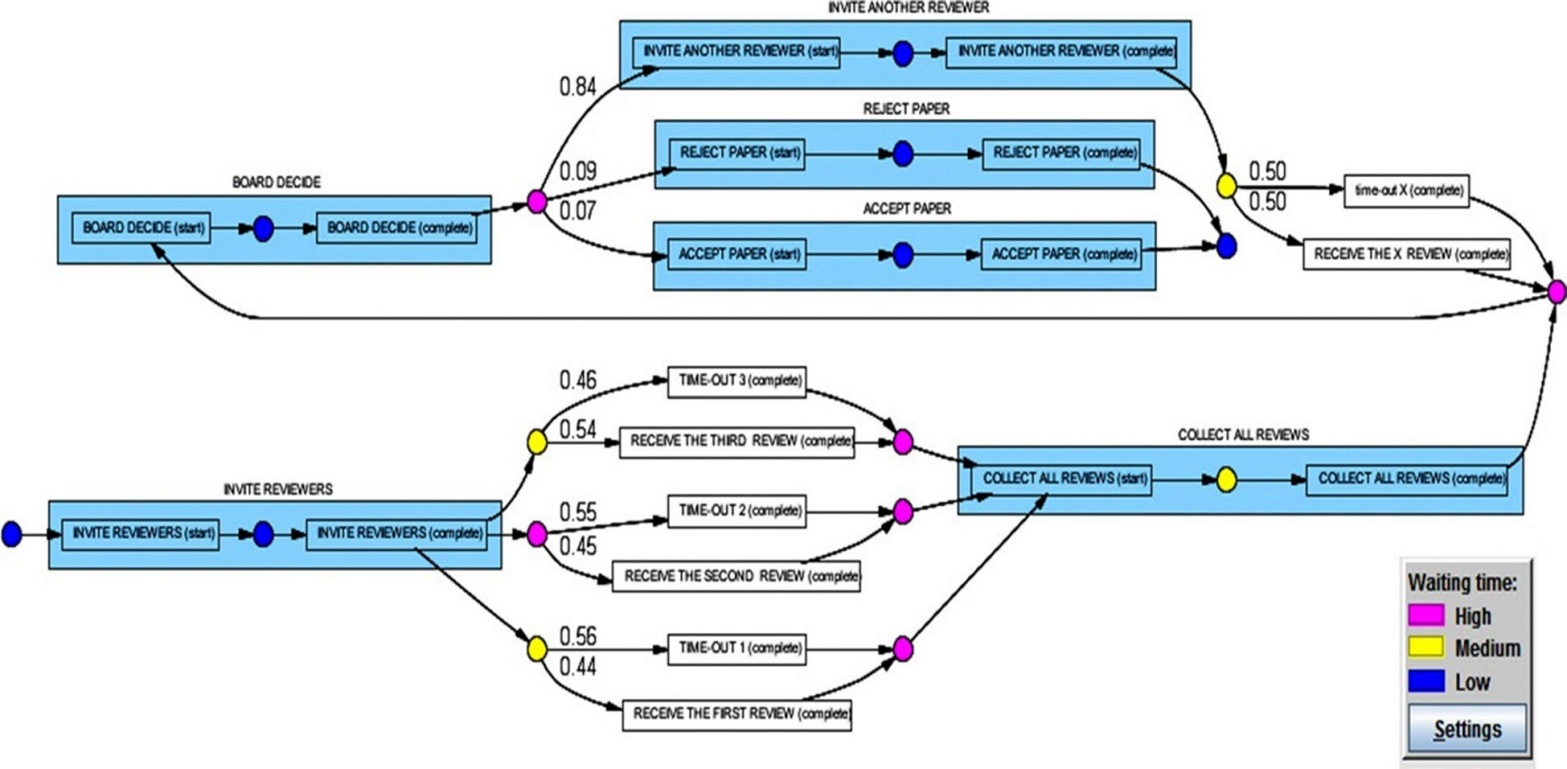
Using the Performance Analysis with Petri Net technique (Aalst 2011) enabled us to investigate the total waiting time during the peer review process. The nodes in *blue* show the tasks performed in less than 7 days (i.e., low waiting/response time). The nodes in *yellow* represent the tasks that were completed between 7 and 21 days (i.e., medium waiting/response time). The nodes in *red* indicate the tasks performed in more than 21 days (i.e., high waiting/response time).

We used the Performance Analysis technique with the Petri Net feature of ProM in order to investigate the *total waiting time* during the peer review process as well. Figure 13 shows information about waiting times during the peer review process for the proceedings of an international conference in Thailand. We categorized the waiting times in terms of High, Medium and Low (Performance Analysis with Petri Net 2009). If a reviewer sends his/her feedback in less than 1 week (7 days), it will be considered as a “*Low*” response time which is shown in the blue color. If a reviewer sends his/her feedback within 7–21 days, it will be considered as a “*Medium*” response time which is shown in the yellow color. And finally, if a reviewer sends his/her feedback after more than 21 days, it will be considered as a “*High*” response time shown in the red (critical) color. By studying the results of the Performance Analysis technique with the peer review Petri net, we realized that in only 45% of the cases was feedback from the Second Reviewer received on time, while in 55% of the cases, the Second Reviewers did not participate in the peer review process until the time-out was reached (i.e., their time and deadline to review the manuscript was over). Interestingly, those 45% of feedback from the Second Reviewers were also received with long waiting times (i.e., high response time shown in the red color). Thus, the reviewing process to collect feedback from the Second Reviewers become too long and ineffective; therefore, the proceedings organizers need to fix this problem for the forthcoming peer-review processes in future. On the other hands, although the feedback from the First Reviewers and the Third Reviewers were received within 7–21 days (i.e., including the reviewers’ comments or timeout) which were fair enough, the organizing committee members wasted a lot of time (shown in red color) to collect and report their feedback. Therefore, in order to increase the efficiency and effectiveness of the peer review process, the conference’s organizers also need to speed up the process of attending to the feedback received from the reviewers in upcoming conferences.

## Conclusions

In this study, we used an event log describing the handling of reviews for the proceedings of an international conference at a private university in Thailand. The authentic event log was originally in Microsoft’s Access database format and was consisted of 87 cases (i.e., number of submitted manuscripts) and 3267 events (i.e., total number of tasks performed throughout the peer review process). Knowing that that *ProM 5.2* only receives the input data in MXML-formatted event logs, we used the functions *addPIandAttr* and *returnFieldNamesInArray* (in Visual Basic script) to create four tables with specific structures. These functions were created based on the concept that we needed a table in which each row contained information about a unique process instance as well as additional information for each process instance. Subsequently, *ProMimport* was chosen as a framework to extract a MXML-formatted log from the data source, and an ODBC connection was configured between the data and the personal computer (PC). Since the event log contained sensitive details concerning the authors and their personal information; the event log was deliberately made anonymous in the necessary areas. After successfully converting the data into MXML format, we applied Alpha, Heuristic and Fuzzy process mining techniques (from the *process discovery* class of the process mining analysis tools) in order to discover models and extract information from the peer-review event log. Using the Alpha algorithm, we could investigate the peer review process with respect to the tasks that came before/after other tasks, the tasks that concurrently occurred with other tasks, or the tasks that were duplicated (i.e., loop) in the model. The results showed that although each paper was initially sent to three different reviewers; it was not always possible to make a decision after the first round of reviewing; therefore, additional reviewers were invited. In total, all the accepted and rejected manuscripts were reviewed by an average of 3.9 and 3.2 expert reviewers, respectively. However, when dealing with noisy data, the Alpha algorithm could not produce reliable and robust models—as any frequency information about dependencies of the tasks is not taken into account. Therefore, we used a more sophisticated technique named Heuristic Miner algorithm in order to illustrate and investigate the dependency between different tasks during the peer review process. Our main objective was to create models that were less sensitive to the incompleteness of logs and contain noise. Although the idea of applying Heuristic Miner algorithm was interesting, yet the technique could not properly deal with spaghetti-like structures which contain mixed AND and XOR joints/splits. Therefore, we used Fuzzy Miner technique (as a more sophisticated approach) to overcome these limitations and constraints. When studying the resulting fuzzy model, we realized that the tasks “*Board Decide*”, “*Invite Another Reviewer*”, “*Time*-*Out X*”, and “*Receive The X Review*” were the most significant tasks (during the peer review process of the international conference) allocating 27.52, 23.16, 11.68, and 11.48% of the performed tasks to themselves, respectively. Furthermore, we applied ideas obtained from cartography to build process-model maps based on the Fuzzy Mining algorithm. The result was an animation that produced with a better understanding of what has occurred during the proceedings peer-review process.

As a part of the *process discovery*, we also applied three metrics with the purpose of building graphical social network models in terms of: (a) Handover of Task, (b) Similarity of Task, and (c) Working Together. The main idea was to identify different groups and communities of the conference’s committee members and organizers (clusters) through different Social Network Miner techniques.

Consequently, using LTL approach, Conformance Checking, and Performance Analysis with Petri Nets (from the *conformance checking* class of process mining tools), we could assess the fitness of the models by detecting discrepancies between the authentic data and the pre-defined master model. The results showed that 9 blocks of activity in authentic peer review event log were not compatible (not fit) with the activities in the pre-defined Petri net model (i.e., master model). Moreover, 5 activity traces were not correctly enabled in the authentic event log. Also, 7 activities were missed in the authentic event log compared with the pre-defined master Petri net model. In other words, the resulting Petri net model and the real peer review event log were not a complete fit and compatible with each other at a few points and nodes. Moreover, only in 45% of the cases a feedback from the second reviewer was received on time, while in 55% of the cases, the second reviewers did not participate in any peer review process until the time-out was reached. On the other hand, dealing with the feedback (comments) received from the first and the third reviewers; the conference committee members and the organizers did not attend to those feedback/comments in a timely manner.

## Endnotes

^a^For more information about ProM and Process Mining visit the website:http://www.processmining.org/.

^b^For more information about ProMimport visit the website: http://www.promtools.org/promimport/.

^c^For a detailed description of the α-algorithm and a proof of its correctness please check the link: http://en.wikipedia.org/wiki/Alpha_algorithm.

^d^For more information about basics of the Social Network Analysis techniques read the book titled “Social Network Analysis Theory and Applications” in the link below: http://train.ed.psu.edu/WFED-543/SocNet_TheoryApp.pdf.

^e^For more information about the Conformance Checker tool visit the link below: http://www.processmining.org/online/conformance_checker.

^f^For further information about the Performance Analysis with Petri Nets check the link below: http://www.processmining.org/online/performanceanalysiswithpetrinet?s[]=performance&s[]=analysis.

^g^For detailed information about the functions please check the “manual” provided in the link below: http://www.processmining.org/promimport/%20tutorials.

^h^For more information about the Similarity Coefficient please visit and read our article titled “Prom: Analysis of Social Network in students registration system” in 10th International Conference of ICT and Knowledge Engineering (ICT & Knowledge Engineering) published in IEEE Xplore.
